# A Reinforcement Learning Based Dirt-Exploration for Cleaning-Auditing Robot

**DOI:** 10.3390/s21248331

**Published:** 2021-12-13

**Authors:** Thejus Pathmakumar, Mohan Rajesh Elara, Braulio Félix Gómez, Balakrishnan Ramalingam

**Affiliations:** Engineering Product Development Pillar, Singapore University of Technology and Design (SUTD), Singapore 487372, Singapore; pathmakumar_thejus@mymail.sutd.edu.sg (T.P.); rajeshelara@sutd.edu.sg (M.R.E.); braulio_felix@mymail.sutd.edu.sg (B.F.G.)

**Keywords:** audit robot, path planning, reinforcement learning, cleaning-auditing

## Abstract

Cleaning is one of the fundamental tasks with prime importance given in our day-to-day life. Moreover, the importance of cleaning drives the research efforts towards bringing leading edge technologies, including robotics, into the cleaning domain. However, an effective method to assess the quality of cleaning is an equally important research problem to be addressed. The primary footstep towards addressing the fundamental question of “How clean is clean” is addressed using an autonomous cleaning-auditing robot that audits the cleanliness of a given area. This research work focuses on a novel reinforcement learning-based experience-driven dirt exploration strategy for a cleaning-auditing robot. The proposed approach uses proximal policy approximation (PPO) based on-policy learning method to generate waypoints and sampling decisions to explore the probable dirt accumulation regions in a given area. The policy network is trained in multiple environments with simulated dirt patterns. Experiment trials have been conducted to validate the trained policy in both simulated and real-world environments using an in-house developed cleaning audit robot called BELUGA.

## 1. Introduction

Cleaning is one of the indispensable factors associated with everyday life. It is a progress indicator for a plethora of organizations spanning from a small living space to a nation’s development index [1,2]. The importance of cleanliness accelerates the global cleaning industry to a steep annual growth of 10% by 2026, and valued more than 46 Billion USD in 2020 [3,4]. The significance of the cleaning industry acted as a catalyst for bringing bleeding-edge technologies in the field of domestic and professional cleaning [5,6,7,8,9,10]. The rise of cleaning robots is an excellent example of technological advancement in the field of the cleaning industry. The success of robot-based automated cleaning has pushed the boundaries of cleaning robots even further towards the adaptation of novel strategies for mechanism, control, perception, etc. For example, the work mentioned in Prabakaran et al. [11] reports the studies on shapeshifting techniques for a self-reconfigurable floor cleaning robot. Muthugala et.al. developed novel adhesion-awareness strategies for vertical glass panel cleaning robot [12] by exploiting the fuzzy inference system for achieving control goals. An adaptive floor cleaning strategy has been introduced in work reported in Sivanantham et al. [13], where authors used the human density surveillance factor as the driving factor for the floor cleaning strategy. The study mentioned in Chang et al. [14] reports the development of an efficient automatic recharging mechanism for cleaning robots. In the research work mentioned above, the authors used neural networks for location estimation and infrared spots to guide the robot for successful docking. Pathmakumar, et al., proposed a novel cleaning path-planning strategy for hydro-blasting robots, where the authors exploited genetic algorithms for determining the functional footprint that aids an optimal coverage path-planning strategy [15].

Despite the technological advancement in automated cleaning and cleaning robots, few crucial research problems are left unaddressed. One is the performance evaluation of the cleaning robot based on the quality of cleaning it delivers and to benchmark the quality of cleanliness. However, the aforementioned problems can be fundamentally addressed by defining a method for auditing cleanliness. At present, a primitive research focus is given in auditing cleanliness. For example, the work reported in Diab-El Schahawi et al. [5] discusses the possibilities, advantages, and limitations of the usage of UV disinfection robots to reinforce the cleaning technology amidst the COVID-19 pandemic. The research article mentioned in Giske et al. [16] reports the effectiveness of robot-based cleaning in the fish processing industry. Furthermore, the work mentioned in Lewis et al. [17] details the usage of ATP (Adenosine Triphosphate) bioluminescence techniques for assigning a benchmark value for cleanliness. Systematic procedures for performing cleaning validation are presented in the article mentioned in Asgharian et al. [18]. The above mentioned article suggests guidelines for cleaning validation and report generation in the pharmaceutical industry. A validation assessment report for cleaning disinfection in a hospital setting is presented in Malav and Saxena [19]. This article explains the inspections and swab analysis of the surfaces and appliances of concern, followed by laboratory analysis. Currently, the effort towards evaluating the quality of cleaning is targeted only to specific sectors, like hospitals, food processing, and pharmaceutical industries, etc. [20,21]. However, cleaning is a routine that cuts across all levels of domestic and industrial sectors. Hence, the strategy for cleaning auditing has to be adaptable to a broad spectrum of domains beyond specific sectors. Besides, the reported techniques for the assessment of cleanliness remain manual, less scalable, and laborious. Hence, there is an apparent necessity for a cleaning-auditing strategy that addresses the limitation of prevailing methods. From the prior art, it is evident that automated cleaning lacks an effective solution for auditing despite its technological advancement. Leveraging upon the advances in robotic technology, developing an autonomous cleaning-auditing robot is an effective solution to address the problem. The primary footstep to address this problem was reported in our previous research work mentioned in Pathmakumar et al. [22]. The above mentioned research work introduces a framework for cleaning-auditing using an autonomous mobile robot with a sample audit sensor as a critical payload. The sample audit sensor audits over a small region and assigns an audit score proportional to the extent of cleanliness (also known as sample-auditing). The robot does the auditing for a larger area through multiple sample-auditing in different locations autonomously (also known as space-auditing).

This article proposes a new strategy for autonomously auditing a given area using an cleaning-auditing robot. The proposed system exploits Deep Reinforcement Learning methods and guides the robot towards a region with a higher possibility for dirt accumulation. Thus, the proposed method makes the robot capable of performing auditing entirely driven by its experience. The rest of the manuscript is organised as Section 2 detailing works related to the proposed approach; Section 3 gives overview of the proposed approach, followed by Section 4 which provides formal definitions of system modeling, Section 5 reports strategy for training of the modeled system. The systematic validation of the proposed approach through simulations, experiments, and interpretation of results are given in Section 6, followed by the conclusion of our findings and future work in the Section 7.

## 2. Related Works

In robotics, application of reinforcement learning (RL) is a topic that is given significant research importance [23,24,25,26,27]. The application of Reinforcement learning is mostly used for solving challenges in perception, navigation, and control problems. For instance, reinforcement learning is used for solving complex locomotion tasks for a multi-joint snake-like robot, where a model-free RL algorithm is used to map visual observations, and the state of the joints [28]. The approach mentioned above used the proximal policy optimization (PPO) [29] for model training. A two-stage RL approach is proposed as a solution for collision avoidance in UAV under imperfect sensing circumstances [30]. In the aforementioned work, the implementation of policy training for a collisionless path from noisy local observations is discussed. Deep deterministic policy gradient (DDPG) was used in the above mentioned work [31]. A three-layer deep reinforcement learning architecture that enables goal generation, perception, and planning is given in discussed in Mousavi et al. [32]. The work mentioned also discusses the hierarchical training of planners and classifiers with policy gradient algorithm implemented in actor-critic style. A novel ultrasound image-driven motion planning was introduced to a robot manipulator, where the Double Deep Q-Network (DDQN) approach is utilized for achieving a navigation accuracy of 82.91% [33]. Possibilities of a cost-efficient navigation strategy using depth camera with limited Field of View (FoV) explored in Choi et al. [34]. In the abovementioned research work, authors have done an empirical analysis of FOV upon the efficiency of RL agent and formulation of strategies to improve agent’s performance in real-time. Work mentioned in Pfeiffer et al. [35] reports the usages of reinforcement learning for map-less navigation in mobile robots. The work mentioned above details the use of an end-to-end neural network that maps the raw sensor data to motion commands to the mobile robot. Besides solving complex perception and navigation problems, reinforcement learning is also widely used for task-based robot applications. For example, an A3C algorithm based for search and rescue operation is reported in Niroui et al. [36], where authors modified the state of the art frontier exploration with deep reinforcement learning methods for efficient area exploration in a mobile. Similarly, strategy has been used for achieving a UAV-based search and rescue operation in Zuluaga et al. [37]. Again, A multi-robot collision-free exploration strategy is discussed in work mentioned in Hu et al. [38]. The authors used novel dynamic Voronoi partitions for robot exploration and a model-free deep deterministic policy gradient (DDPG) algorithm for collision avoidance. Similarly, DDPG based reinforcement learning methods are used for search and rescue operation in unmanned areal vehicles [39].

The proposed approach treats the robot as a reinforcement learning agent, and it learns and generalizes the dirt distribution pattern from multiple environments during its training phase. Then, based on the policy it learned, the robot chooses the locations it has to go to in the rollout phase. The proposed method enhances the space-auditing procedure in the cleaning-auditing framework [22], since its performance depends on training data which can be simulated easily. The overall objective of this research work is subdivided into four components.

1. Model the autonomous auditing as a Markov decision process (MDP).
2. Train the model with representation of indoor environments with manually assigned dirt distributions.
3. Evaluate the learned policy on multiple simulation environments.
4. Using in-house developed BELUGA cleaning audit robot, evaluate the performance of the proposed strategy.

## 3. System Overview

**Figure 1:**
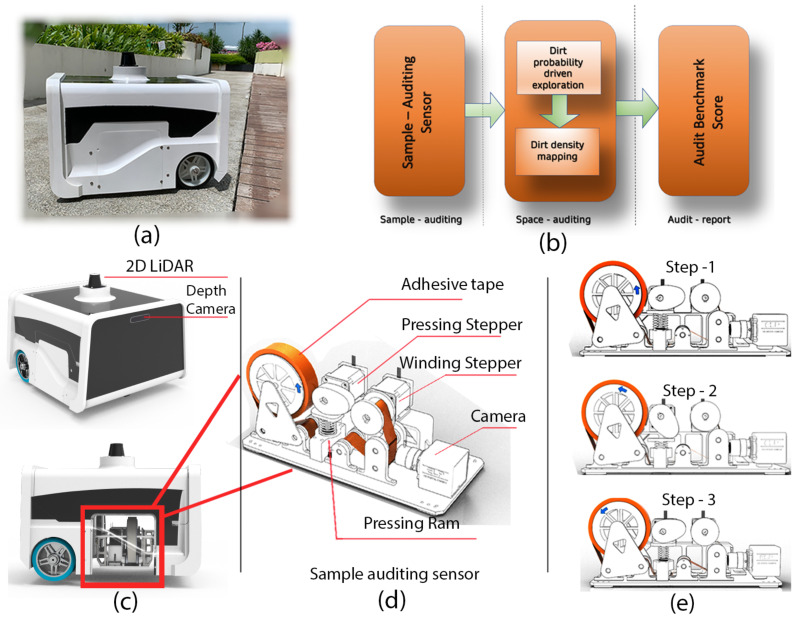
The components associated with autonomous cleaning-auditing, the *BELUGA* robot (**a**), the cleaning-auditing framework (**b**), robot with navigation sensors and sample audit sensor (**c**), components inside the sample audit sensor (**d**), and the steps involved in sample auditing process (**e**).

To start with the proposed strategy for auditing, the background of the audit robot mentioned in Pathmakumar et al. [22] (also known as BELUGA robot) for completeness and clarity. The Figure 1 shows the overview of components and procedures involved in cleaning auditing. The robot is designed for auditing the cleanliness of a region autonomously (Figure 1a). The BELUGA robot runs cleaning-auditing framework shown in Figure 1b. The BELUGA platform has a three-point contact differential drive mechanism and carries visual and inertial sensors for autonomous navigation Figure 1c. Besides, a sampling audit sensor is a crucial component onboard the BELUGA robot aiding cleaning-auditing Figure 1c. The sample audit sensor evaluates the dirtiness of a small area (sample area) by a “touch and inspect” analogy and assigns a sample audit score for the location. The sensor uses adhesive tape to extract the dirt from the floor with the help of the co-coordinated movement of stepper motors associated with it. Figure 1d shows a sampling cycle, which is comprised of three steps: (1) pressing action that makes the adhesive tape stick on the floor with adequate pressure to extract the tiny dirt particles to its sticky surface; (2) The winding of adhesive tape by winding stepper motors to move the adhesive tape surface to the field of view (FoV) of a camera; and finally, (3) capturing of the images of the adhesive tape surface for dirt analysis. The extent of dirtiness of the sampled region is estimated using structural similarity index (SSIM) analysis [40] and saliency-based dirt detection [41,42] using the embedded computer on the BELUGA platform.

The sampling audit sensor inspects a sample area (2 cm × 2 cm) and provides an audit score (in the range of 0–1) that defines the extent of dirtiness. This method of estimating the uncleanness of a given area is called sample auditing in auditing framework shown in Figure 1b. Performing a repeated sampling auditing for the entire region can estimate global dirt distribution in the robot’s area of operation. This process of repeated sampling auditing is called space auditing in the auditing framework shown in Figure 1b.

**Figure 2:**
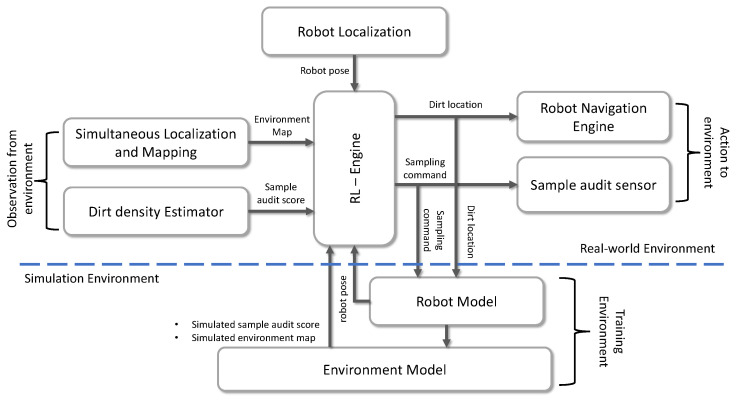
The overview of RL based dirt exploration strategy for autonomous cleaning-auditing robot.

Performing sample auditing for a large area is not efficient and time-consuming. Nevertheless, it is more practical to do sampling only in regions where the possibility of accumulating dirt is higher. In addition, to execute the sampling strategy, the global dirt distribution pattern of the environment has to be known prior, making the dirt sampling problem more challenging to address. However, RL can be used to solve the problem mentioned above and formulate a dirt exploration strategy. The Figure 2 shows the overview of the proposed dirt exploration strategy that can be implemented in an autonomous mobile robot with a sampling audit sensor. The central component in the proposed system is an RL-Engine composed of trainable RL models. The input to the RL-Engine can be taken either from a real-world environment or a simulation with models for robot and environment. The dirt exploration strategy is modeled as a model-free RL algorithm executing within the RL engine. The output from RL-engine is the location where the robot needs to go, which is fed into the robot or to a robot model in the simulation. Besides, the RL engine also provides the sampling command that triggers a sampling action by the sensor. The input to the RL-engine will be the map of the environment, the position of the robot, and the sample audit score. The above mentioned inputs to the RL engine are provided by the simultaneous localization and mapping module, dirt density estimator, and robot localization module. The RL-engine training can be done by taking the input from the simulations of the environments where the cleaning auditing has to be carried out. The approach mentioned above for dirt exploration has the following advantages:

1. Simple: Easier to model the problem since an analytical model for dirt distribution is harder to compute.
2. Scalable: The dirt accumulation patterns can be simulated easily for training, and the RL model can be scaled to any environment.
3. Reliable: The system’s performance depends upon the learned policy for selecting actions and less relies upon limiting factors like sensor accuracy, external lighting, etc.

The rest of the sections provide detailed information regarding the RL-algorithms implemented inside RL-engine, simulation environments, strategy adapted for training, and the integration proposed dirt exploration strategy on our in-house developed BELUGA robot.

## 4. Problem Formulation

This section discusses the modeling and formulation of autonomous dirt exploration problem for cleaning-auditing, treating as a deep RL problem. The foremost step is modeling the system as a Markov decision process (MDP) and providing a formal definition of processes and states associated with MDP. We have used proximal policy approximation (PPO) [29], a gradient-based optimal policy approach to determine the best action to be taken by the RL agent. The action selection by the agent is made based on the output of a neural network. We considered RL agent, as the BELUGA robot with a sample audit sensor. The map of the environment, dirt profile, and position of the robot are the observations made by the robot from the environment. The observations are fed into the networks to get the probability distribution of actions and value. The detailed descriptions on the formal definition of MDP and PPO are given below.

### 4.1. Markov Decision Process (MDP)

The dirt exploration is treated as a discreet time finite MDP. MDP is defined by a tuple $\langle S,A,R,P\rangle$ where, S is the state space, A is the action space and *R* is the reward function respectively. P is the state transition probability function P:S×A×S→R. We consider the robot as an RL agent that make observation from different states $s_{t}\;\epsilon \;S$. For a time step *t* the agent performs an action $a_{t}\;\epsilon \;A$. The environment assigns a reward $r(s_{t},a_{t})\;\epsilon \;R$ corresponding to the action performed by the agent in every state. The procedure mentioned above will be restarted when agent progress to the next state $s_{t+1}$. The environment is the region of operation of the robot. In the scenario of concern, the environment consists of profile of static obstacles and dirt distribution on the floor. The sate of the robot at any time step *t* can be represented as a vector, as given in Equation (1).

(1) $$s_{t}=\left\{\begin{array}{c} l_{t},M \end{array}\right\}$$

where $l_{t}$ is the position of the robot and *M* is the obstacle profile, which is a binary representation of obstacles (0—free space and 1—obstacle) in a 2D-map. The action performed by the robot is visiting location in the map and performing a sampling action. The modeled action space A is given in Equation (2)

(2) $$A=\left\{\begin{array}{c} p,S \end{array}\right\}$$

where, $p:p\in \left\{\begin{array}{c} p_{1},p_{2},p_{3}\ldots p_{i} \end{array}\right\}$ represents the location for sampling and $S:S\in \left\{\begin{array}{c} S_{1},S_{2},S_{3}\ldots S_{i} \end{array}\right\}$ is the state of the sample audit sensor. In this work, we defined four locations for the robot and two states for sampling audit sensor for defining the action set. The Table 1 provides the list of locations and sensor states used.

The agent (robot) gains a reward from the environment based on the action performed in a state. The reward is determined by a reward function that can be represented as a real-valued function that maps all the state-action pairs to a real number, defined as R:S×A→R. In the given scenario, the reward is decided based on, (1) whether the sampling is done in locations where dirt is accumulated (2) sampling is done only in a unique location where sampling is not done before, (3) sampling is done in a place where possibilities of collision with an obstacle is less. Hence, the reward function can be represented as given in Equation (3):

(3) $$R=R_{dirt}+R_{unique}+R_{collision}$$

The parameter $R_{dirt}$ is the rewarding factor for the agent for a sampling action done in a location where dirt exists. If the output of the sample audit score given by the sensor σ is greater than zero, $R_{dirt}$ is assigned as reward Equation (4).

(4) $$R_{dirt}=\left\{\begin{array}{cc} 1, & \sigma >0\\ 0, & \mathrm{otherwise} \end{array}\right.$$

The parameter $R_{unique}$ is the punishing factor for the agent if the sampling is done in a location which is already sampled. The robot keeps record of all the locations visited in a data vector $p_{sampled}$. The parameter $p_{t}$ is the location of the robot at the current time step *t*. The parameter $R_{unique}$ is assigned as reward if the sampling is done in a unique location as given in Equation (5).

(5) $$R_{unique}=\left\{\begin{array}{cc} 1, & p_{t}\notin p_{sampled}\\ -1, & \mathrm{otherwise} \end{array}\right.$$

Similarly, $p_{collision}$ the set of all points with high chances of collision and a reward of $R_{collision}$ is assigned to the robot for an attempt to move to a location in $p_{collision}$ as given in Equation (6).

(6) $$R_{collision}=\left\{\begin{array}{cc} -10, & p_{t}\notin p_{collision}\\ 0, & \mathrm{otherwise} \end{array}\right.$$

The goal of the agent is to learn a behavior that maximizes the reward obtained. This behavior of the agent is known as policy π such that π:S×A→R. The policy is a probability distribution of an action taken given in a state represented as π(a|s). However, for a deterministic case, the policy function can be defined as π(s), a function that maps elements in state space to action, which can be represented as π:S→A. The agent have to find an optimal policy to choose the action that maximize the reward, and it is achieved using Proximal Policy Optimization.

### 4.2. Proximal Policy Optimization (PPO)

PPO, is similar to the policy gradient (PG) method for reinforcement learning. Unlike the conventional PG method the training of a PPO agent is less challenging. This is because, PPO has less hyperparameters, and it employs the principle of importance sampling to ensure the data-efficiency during training. The objective function of a PPO given in Equation (7):

(7) $$J^{PPO}=\hat{E}\left[min\left(r_{t}\left(\theta \right)\hat{A}\;\;,\;\;clip\left(r_{t}\left(\theta \right),\;\;1-\epsilon ,\;\;1+\epsilon \right)\hat{A}\right)\right]$$

The $J^{PPO}$ is also know as the PPO’s clipped surrogate objective function. The $r_{t}\left(\theta \right)$ (policy ratio) is the ratio of action probabilities for the present and previous policies as shown in in Equation (8)

(8) $$r_{t}\left(\theta \right)=\frac{\pi_{\theta }\left(a_{t}|s_{t}\right)}{\pi_{\theta_{old}}\left(a_{t}|s_{t}\right)}$$

In Equation (8), $\pi_{\theta }$ is the action probability under the current policy and $\pi_{\theta_{old}}$ is the action probability under the old policy used for the rollout. When the value for $r_{t}\left(\theta \right)\leq 1$ implies, the action under the current policy is more probable, and a value for $r_{t}\left(\theta \right)$ in between 0 and 1 shows, action under the current policy is less probable than the previous one. However, a larger value for $r_{t}\left(\theta \right)$ is not advised since it can results in over sized gradient steps. Hence, in the the surrogate objective (Equation (7)), the $r_{t}\left(\theta \right)$ ratio has been clipped in a range of 1−ϵ and 1+ϵ. The $\hat{A}$ in Equation (7) is estimated using general value estimation [43]. The ϵ is one of the hyperparameter for PPO.

### 4.3. Network Architecture

**Figure 3:**
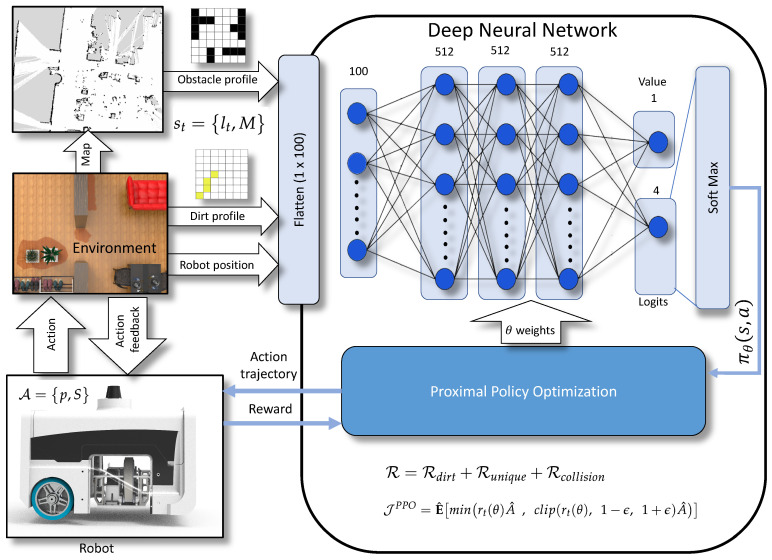
The reinforcement learning architecture based on Proximal Policy Optimization.

The Figure 3 shows the network structure implemented for the PPO. The action probability $\pi_{\theta_{old}}$ and values are estimated using a neural network that is composed of one input layer and two outputs. The input to the neural network is the representation of obstacle profile (7×7), locations of the dirt identified via sample auditing (7×7), and robot position in 2D coordinates (2×1) stored in tensors. The input tensors are flattened to 100×1 and fed into the input layer of the neural network. The network comprises of three hidden layers of size 512×1 sizes each. The neurons in all stages are activated using tanh [44] activation function. The first neuron in the output layer is taken for the value estimation, and the rest carries the logits. An additional softmax layer converts the logits to the corresponding probability distribution. The Equation (7) is used as the loss function for training the network and θ is updated by maximizing the objective function $J^{PPO}$. The best action from the distribution is rolled out to the robot model as action trajectories, which are the waypoints that the robot has to visit next. The Algorithm 1 shows the pseudo-code for implementation of PPO mentioned in [29].
**Algorithm 1** Pseudocode for PPO implementation [29]1:**while**i=1,2,3…**do**2:      **while** action=1,2,3,…N **do**3:            Run policy $\theta_{old}$ for *T* time-steps4:            a←max(action_array)5:            Compute advantage estimates ${\hat{A}}_{1};{\hat{A}}_{2},\ldots {\hat{A}}_{T}$6:      Optimize $J^{PPO}$ with *K* epochs and M≤NT7:      $\theta \leftarrow \theta_{old}$

## 5. Training and Validation

**Figure 4:**
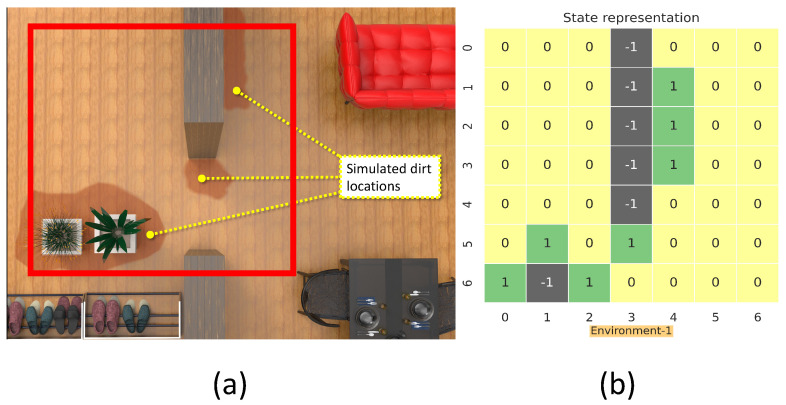
The simulation environment 1 (**a**), and 7×7 reduced environment representation (**b**).

**Figure 5:**
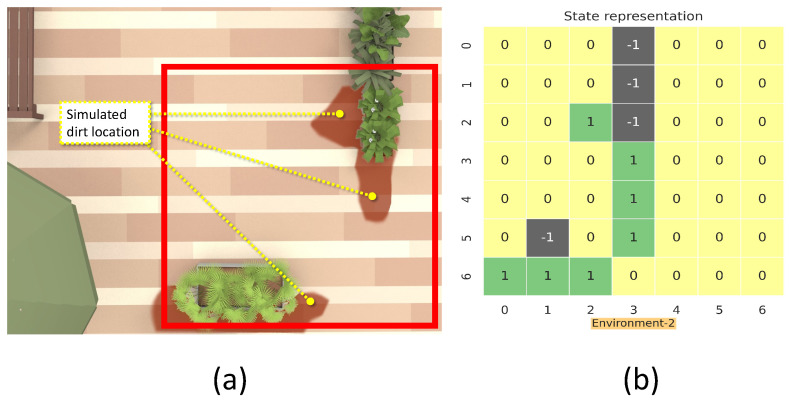
The simulation environment 2 (**a**), and 7×7 reduced environment representation (**b**).

**Figure 6:**
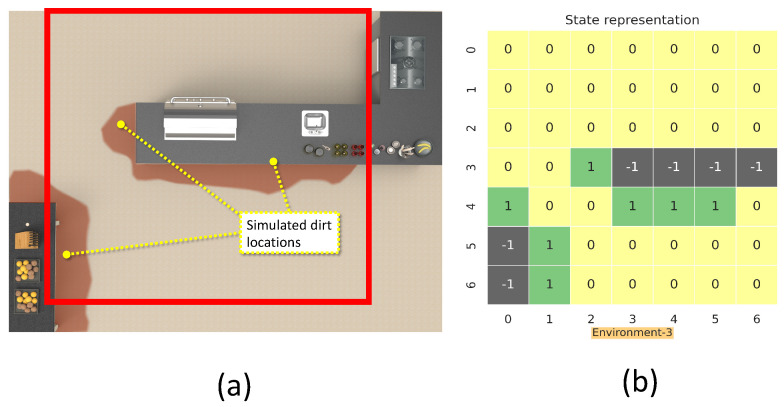
The simulation environment 3 (**a**), and 7×7 reduced environment representation (**b**).

**Figure 7:**
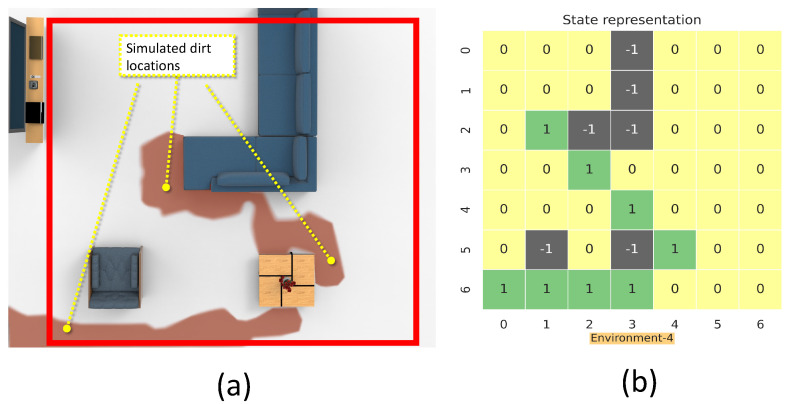
The simulation environment 4 (**a**), and 7×7 reduced environment representation (**b**).

This section details the training and validation for the modeled RL approach for dirt exploration. We simulated four different environments with obstacles, closed walls, and dirt regions. We chose a square space in each environment and randomly distributed the dirt around the walls and obstacles on the map. This map is subdivided into 49 square zones of size 1 m × 1 m. The environment is represented using a 7 × 7 matrix, where the elements represent the possible locations for sampling with dirt regions and possible collision regions. The 7 × 7 reduced representation of the environment is used for training the system. The reduced environment representation is used to make the training process faster with minimal computation. For implementation, we used Gazebo for modeling the environments, and a SLAM algorithm-generated map has been downsampled to generate the reduced environment representation. Figure 4, Figure 5, Figure 6 and Figure 7 shows the 7×7 reduced representation of the environments used for training.

### Policy Training and Evaluation

The training and rollout of the model is implemented using RLLib [45,46] with Pytorch [47] and OpenAI Gym [48] toolkit. RLlib is an open-source framework developed based on Ray [49], that allows optimized implementation of reinforcement learning models using scalable and unified application programming interface (API).

**Figure 8:**
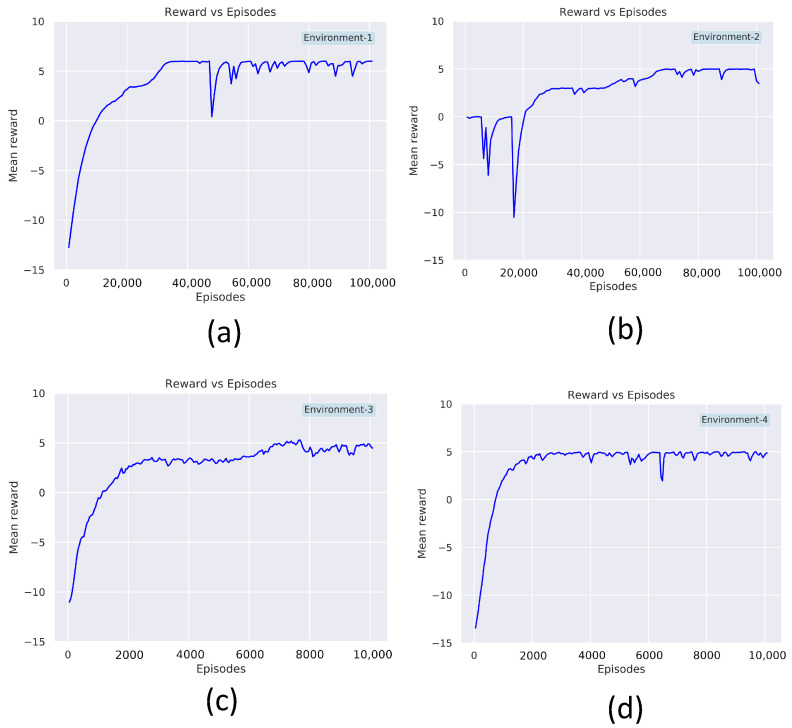
Mean reward vs. number of epochs for (**a**) environment 1, (**b**) environment 2, (**c**) environment 3, and (**d**) environment 4.

The nested parallelism and resources-aware computation property of RLlib allows an efficient training and evaluation of the model. The Table 2 shows the list of hyperparameters used for the PPO implemented using RLLib APIs. The model training is done in an Ubuntu 20.04 running machine with intel core-i7 CPU and RTX-3080 GPU from NVIDIA. The hyperparameters are chosen based on observations from multiple trials that resulted in better convergence. The discount factor γ is chosen as 0.99 with a learning rate of 0.001. The degree of parallelism of the training process is determined by the parameter num_workers, set to 20. The num_sgd_iter parameter is set to 16, which decides the number of times the Steepest Gradient Descend (SGD) has to be done per batch and to enable the experience reply. The parameters clip_param (ϵ) is associated with the surrogate loss function of PPO (Equation (7)). The learning_starts is an initialization parameter that decides the number of steps the model has to sample before starting the learning process. The termination criteria for each episode is either to gather a maximum reward of 10. The maximum step size per episode is kept as 35. The training is done on environment-1 to environment-4. The Figure 8 summarizes the learning curve for training on environment-1 to 4. Environment-1 (Figure 8a), took 80,000 epochs to reach the maximum mean average reward of 5. Similarly, environment-2 (Figure 8b), took 80,000 epochs to converge to a mean average reward of 5. In order to reach the same mean average reward of 5 Environment-3 and Environment-4, took 10,000 episodes of training each. After the training phase, the model is rolled out for real-time testing and policy evaluation. The Algorithm 2 describes the procedures involved for testing the trained model in a robot. The Algorithm 2 repeatedly computes the actions for the robot by taking in the observations from the environment as input. The MoveRobot routine in the algorithm communicates to the navigation framework of the robot. The action trajectory from the trained model provides the next waypoint for the robot to clear.

The Algorithm 2 shows the pseudo-code implementation for the rollout framework of the robot. The Algorithm 2 is dependent on both the Ray and Gym libraries. The robot is configured with navigation algorithms running on ROS [50] noetic version. The robot is localized using Adaptive Monte Carlo Localization(AMCL) method. The model is loaded with trained weights. The current observations made on the environment and reduced environment representations are loaded into the Gym APIs. The output of the rollout algorithm is fed into the navigation stack of the BELUGA robot. The robot uses A* global planner [51] and DWA local planner [52] for navigating from one point to another.
**Algorithm 2** Pseudo code for PPO rollout  1:**procedure**Rollout  2:      Load model weights on agent  3:      obs←Currentobservationoftheenvironment  4:      env←Environmentsimulation  5:      **while** done=True **do**  6:            action_array←ComputeAction(obs)  7:            a←max(action_array)  8:            state←env.step(a)  9:            reward←state[“reward”]10:            obs←state[“obs”]11:            done←state[“done”]12:            MoveRobot(a)

## 6. Results and Discussion

The proposed strategy for dirt sampling has been evaluated by performing multiple experiment trials. We have chosen four sets of evaluation trials. Among the four sets of trials, the first pair (trial-1 and trial-2) are done in a simulated environment, and the other two are done in a physical environment. For the simulations, we used Gazebo-ROS framework for setting up the environment and robot. The simulation environment for trial-1 and trial-2 comprised of 10 m × 10 m indoor settings with walls and obstacles. The dirt distribution is simulated by defining the dirt locations alike to the training environment in the map. We modeled a differential drive robot with wheel radius, wheel separation, and footprint size similar to the physical BELUGA audit robot in the Gazebo platform. The effectiveness of the auditing strategy can be evaluated based on the robot’s ability to choose a waypoint where the dirt is accumulated and perform sampling. The locations that the robot visited, and locations sampled by the robot are recorded during the trials. If the robot decides to perform sampling, the success of the decision is determined, if the chosen location already has dirt accumulation. A sampling action is regarded as false-positive sampling if the location selected for sampling doesn’t have dirt accumulation. The result of visiting a location by the robot is classified to three:

1. Success: Robot initiate a sampling action and the location truly have dirt accumulation, or the robot didn’t do a sampling action, and the location have no dirt accumulation
2. Type-I error: Robot initiate a sampling action, and the location have no dirt accumulation
3. Type-II error: Robot didn’t do a sampling action, and the location have dirt accumulation

The accuracy of decision of the robot is determined using equation:

(9) $$Accuracy\;of\;decision=\frac{Total\;number\;of\;success}{Total\;number\;of\;locations\;visited}\times 100$$

The initial location of the robot is chosen in one corner of the map, and the rollout algorithm (Algorithm 2) decides the waypoints for the robot to visit and perform sampling. The notation of $W_{i,j}$ represents, the *j*th location in the *i*th trial.

**Figure 9:**
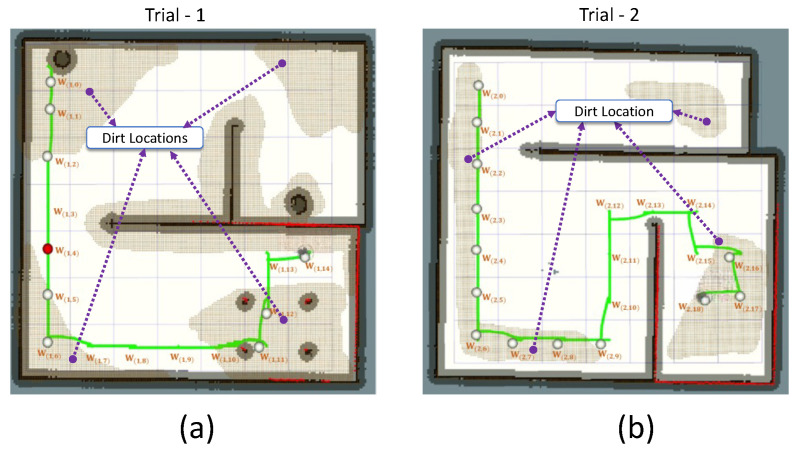
The explored locations by the robot in simulated environments of Trial-1 (**a**) and Trial-2 (**b**) and the path recorded.

The Table 3 and Table 4 tabularizes the observations made from trial-1 and trial-2. In trial-1, the robot cleared 15 waypoint points. Among the cleared waypoints, 11 successes have been registered with a decision accuracy of 73.3%. A total of 9 locations has been sampled for auditing; five have been left unsampled. In the case of trial-2, the robot cleared 19 waypoint points. Eighteen successes have been registered with a decision accuracy of 94.7%. The robot has sampled 13 locations. In trial-1, four type-II errors have been observed. In trial-2, one type-I and zero type-II error have been observed. This is because of the irregularities in dirt distribution patterns in the simulated environment compared to the training environment. The total number of errors reported in the simulated environment is 5 out of 34 locations. However, this shortcoming can be bridged by adding more training data, representing multiple dirt distribution patterns. For trial-1, the robot took a shorter path length to cover than in environment-2. The trial-1 and trial-2 results are satisfactory in terms of the robot’s ability to determine the locations to visit and perform sampling. Figure 9 shows the robot’s exploration path and locations visited in trial-1 and trial-2.

**Figure 10:**
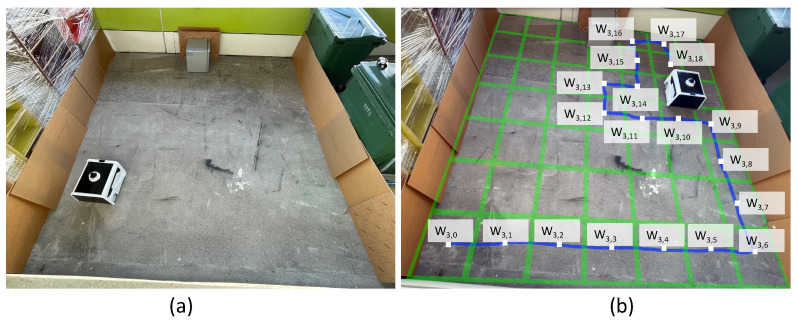
The environment setup for trial-3 (**a**), and the approximate path traversed and locations visited by the robot (**b**).

**Figure 11:**
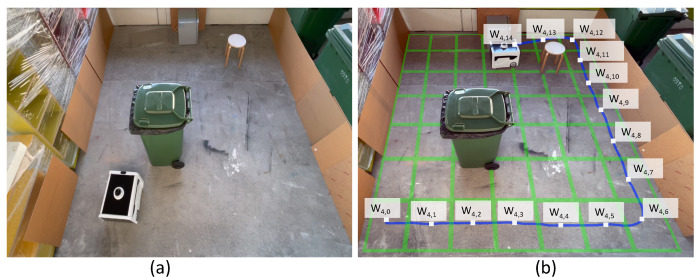
The environment setup for Trial-4 (**a**), and the approximate path traversed and locations visited by the robot (**b**).

Beyond the simulation environments, the model’s effectiveness is evaluated in the real world by integrating the model to the physical BELUGA robot. Unlike the simulation environments with artificially modeled dirt distribution, choosing a real-time environment assess the robot’s ability to select naturally formed dirty locations with the help of a sampling audit sensor. Besides, real-time testing of the rollout algorithm evaluates the shortcomings in the practical implementation of the proposed system. Since the dirt distribution is not known prior, the success and error of a sampling action is determined based on the data from the sample auditing sensor. A pair of evaluation trials (trial-3 and trial-4) are conducted in a real-world environment with BELUGA robot, where a 5.1 m × 5 m section of a broad passage is taken as the region of operation of the robot. However, the mode of operation of the robot remains the same as trail-1 and trial-2. The maximum speed of the robot is capped at 0.25 ms${}^{-1}$. The goal tolerance of the robot is fixed as 0.4 m in x and y positions and 0.2 rad in orientation. The navigation parameter selection is made based on observation of the robot’s accuracy in localization. A 5.1 m × 5 m section of the passage is enclosed with obstacles to create a closed map and localization of the robot is done (Figure 10a and Figure 11a). The passage had dirt in various scales due to the natural dirt accumulation. Hence, during trial-3 and trial-4, we haven’t introduced any artificial dirt distribution. We have used a trash can, wooden stool, and metal box as obstacles in both experiment trials. Comparing trial-3 and trial-4, we have introduced more obstacles than trial-3 to test the robot’s behavior in the dense and less-dense conditions of static obstacles in the environment. The robot mapped both environments separately, and localized before executing the RL-rollout algorithm to initiate the dirt exploration. The Table 5 and Table 6 summarizes the observations made from the trial-3 and trial-4. Since the ground truth of dirt distribution in a real environment is unknown, the data from the sample audit sensor on the robot is used to determine sampling success. A reading above 0.25 by the sensor is regarded as a dirt location and less than 0.25 as a clean location. In trial-3, the robot cleared 19 waypoint points. Among the 19 cleared waypoints, 14 successes have been registered with a decision accuracy of 73.68%. A total of 11 locations have been sampled for auditing; eight have been left unsampled. In the case of trial-4, the robot cleared 15 waypoint points, and Twelve successes have been registered with an exploration accuracy of 80.0%. The robot has sampled 11 locations, and 4 locations left unsampled. In trial-3, two type-II errors and three type-I error have been observed. In trial-4, one type-I and two type-II errors have been observed. Figure 10b and Figure 11b illustrates the approximate path traversed by the robot during the dirt exploration and waypoints.

**Figure 12:**
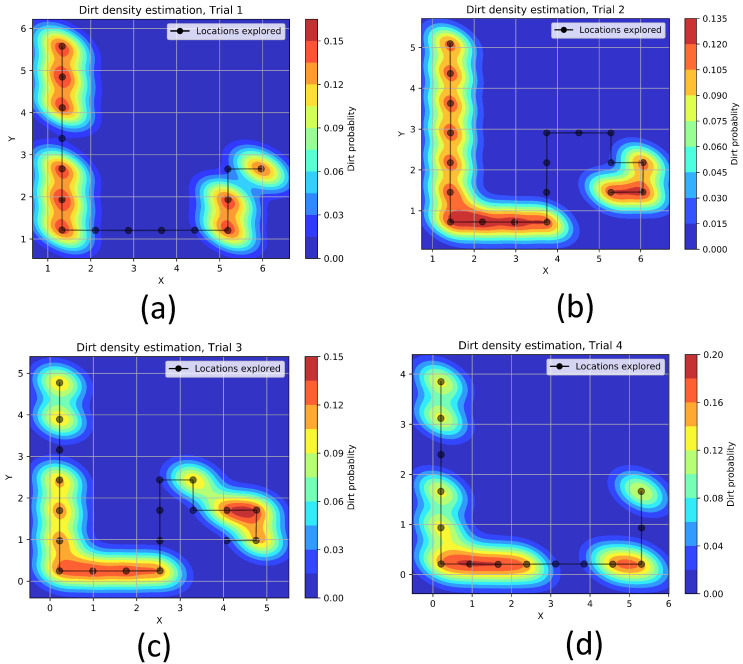
The explored path and estimated dirt density hot-spots in trial-1 (**a**), trial-2 (**b**), trial-3 (**c**) and trial-4 (**d**).

The Figure 12 shows the approximated dirt-density hot-spots in trial-1 to trial-4 using kernel density estimation. We can observe from the experiment trials that the trained model could guide the robot towards the region with dirt accumulations. However, in experiment trials, we observed false positives in both simulated environments and real-world environments. Besides, there are locations where the robot explored but skipped the sampling. This is because of the difference in dirt accumulation patterns in training and testing environments. However, this can be bridged by introducing more variations in dirt accumulation patterns during the training phase. Currently, the training phase only considers fixed and continuous dirt accumulation patterns. However, stochastic modeling of dirt patterns in the training phase would make the system more accountable for randomness in real-world dirt patterns.

One of the shortcomings observed is robot failed to explore all the dirt locations prevailing in the environment. This is because of two reasons; one is robot reaches maximum reward before it explores the all dirt region, and the other is the dissimilarities in training and validation environments.

One of the classic dirt exploration strategies for cleaning auditing is our previous work [22], where the frontier exploration algorithm is modified for dirt exploration. The observed shortcoming of the approach mentioned above was the high chances of false positives. This is because the probable dirt region detected by the robot was dependent on the changes in ambient lighting and floor pattern. However, the proposed method has an upper edge over the method mentioned above since the algorithm’s effectiveness can always be enhanced by introducing more training sequences. Hence, the limitation of sensor accuracy is less affected in the proposed system than the previously reported dirt exploration strategy for cleaning auditing robots. For a cleaning auditing robot, the decision for sampling is as critical as visiting a dirt accumulated region. The proposed system yields the decision to perform sampling after visiting a waypoint that brings completeness to dirt exploration compared to the classical dirt exploration strategy for an audit robot.

## 7. Conclusions and Futureworks

This paper proposed a reinforcement learning-based dirt exploration strategy for a cleaning-auditing robot in an autonomous fashion. The robot-based cleaning-auditing is done with the help of a sample-audit sensor, which is the key-payload of the robot. The proposed exploration strategy provides waypoints closer to the probable dirt accumulated area based on the robot’s learned experience. The audit robot is treated as a reinforcement learning agent, and the exploration problem is modeled as a discreet Markov decision process (MDP). The Proximal Policy Optimization (PPO) on-policy algorithm is used to train the reinforcement learning agent. The model is trained in different simulated environments with dirt distribution, and the trained model is clearly evaluated. The proposed method is validated by conducting experiments in both virtual and real-world environments by using an in-house developed BELUGA audit robot. The future work of this research will be focusing on:

1. Establishing an exhaustive open-source dirt distribution dataset to enhance the training and improvements of the current method
2. Chemical and microbial analysis in sample auditing
3. A comprehensive comparative study on different on-policy algorithms in context of cleaning auditing
4. Multi-agent reinforcement learning for dirt-exploration strategy

## Figures and Tables

**Table 1:** The list of actions done by the robot *p*, and audit sensor state *S*.

*p*	Waypoint in Robot Coordinates	Action Taken by the Robot
$p_{0}$	(0,0)	Robot stay in the current position
$p_{1}$	(0,0.5)	Robot move to left
$p_{2}$	(0.5,0)	Robot move forward
$p_{3}$	(0,−0.5)	Robot move right
***S***	Sensor state	
$S_{0}$	Sensor in idle state	
$S_{1}$	Sensor performs sampling	

**Table 2:** The list of hyperparameters used for training PPO.

PPO-Hyperparameters	Value
discount factor γ	0.99
learning rate $l_{r}$	0.001
mini batch size	128
num_workers	20
num_sgd_iter	16
clip_param	0.2
learning_starts	$10^{4}$
buffer_size	$5\times 10^{4}$
train_batch_size	32

**Table 3:** The list of locations visited by the robot in trial-1 and sampling decision taken.

Waypoint	Location on Map	Decision	Result
$W_{1,0}$	(1.33317,5.57795)	Sample	Success
$W_{1,1}$	(1.33591,4.84829)	Sample	Success
$W_{1,2}$	(1.33509,4.11675)	Sample	Success
$W_{1,3}$	(1.33080,3.38704)	Not Sample	Type-II error
$W_{1,4}$	(1.33087,2.66572)	Sample	Success
$W_{1,5}$	(1.32982,1.93444)	Sample	Success
$W_{1,6}$	(1.33186,1.21004)	Sample	Success
$W_{1,7}$	(2.10888,1.204991)	Not sample	Type-II error
$W_{1,8}$	(2.87477,1.20187)	Not sample	Success
$W_{1,9}$	(3.64719,1.20291)	Not sample	Success
$W_{1,10}$	(4.41937,1.20692)	Not sample	Type-II error
$W_{1,11}$	(5.18784,1.20249)	Sample	Success
$W_{1,12}$	(5.19334,1.93656)	Sample	Success
$W_{1,13}$	(5.18933,2.66006)	Not sample	Type-II error
$W_{1,14}$	(5.96312,2.66641)	Sample	Success
		Total path length	10.45537 m
		Total time taken	86 s
		Accuracy of decision	73.3%

**Table 4:** The list of locations visited by the robot in trial-2 and sampling decision taken.

Waypoint	Location on Map	Decision	Result
$W_{2,0}$	(1.42324,5.09438)	Sample	Success
$W_{2,1}$	(1.42660,4.36318)	Sample	Success
$W_{2,2}$	(1.43137,3.6314)	Sample	Success
$W_{2,3}$	(1.43219,2.9083)	Sample	Success
$W_{2,4}$	(1.42612,2.1773)	Sample	Success
$W_{2,5}$	(1.42348,1.4509)	Sample	Success
$W_{2,6}$	(1.43123,0.723682)	Sample	Success
$W_{2,7}$	(2.19640,0.725312)	Sample	Success
$W_{2,8}$	(2.96881,0.718802)	Sample	Success
$W_{2,9}$	(3.74668,0.719092)	Sample	Type-I error
$W_{2,10}$	(3.73843,1.44793)	Not sample	Success
$W_{2,11}$	(3.74293,2.17497)	Not sample	Success
$W_{2,12}$	(3.74063,2.90702)	Not sample	Success
$W_{2,13}$	(4.51069,2.90921)	Not sample	Success
$W_{2,14}$	(5.28431,2.90556)	Not sample	Success
$W_{2,15}$	(5.28709,2.17860)	Not sample	Success
$W_{2,16}$	(6.05995,2.18154)	Sample	Success
$W_{2,17}$	(6.05311,1.45565)	Sample	Success
$W_{2,18}$	(5.28317,1.45127)	Sample	Success
		Total path length	13.41366 m
		Total time taken	93 s
		Accuracy of decision	94.7%

**Table 5:** The list of locations visited by the robot in trial-3 and sampling decision taken.

Waypoint	Location on Map	Decision	Result
$W_{3,0}$	(0.22162,4.77094)	Sample	Success
$W_{3,1}$	(0.21404,3.88575)	Sample	Success
$W_{3,2}$	(0.21513,3.15650)	Not Sample	Success
$W_{3,3}$	(0.22232,2.43131)	Sample	Success
$W_{3,4}$	(0.21724,1.69945)	Sample	Success
$W_{3,5}$	(0.21509,0.97136)	Sample	Success
$W_{3,6}$	(0.21959,0.24172)	Sample	Success
$W_{3,7}$	(0.98596,0.24564)	Sample	Type-I error
$W_{3,8}$	(1.76468,0.24177)	Sample	Type-I error
$W_{3,9}$	(2.53047,0.24148)	sample	Success
$W_{3,10}$	(2.53720,0.970674)	Not Sample	Success
$W_{3,11}$	(2.53619,1.70709)	Not Sample	Type-II error
$W_{3,12}$	(2.53145,2.42771)	Not Sample	Success
$W_{3,13}$	(3.30528,2.43128)	Sample	Type-I error
$W_{3,14}$	(3.30776,1.70053)	Not Sample	Success
$W_{3,15}$	(4.07132,1.70240)	Sample	Success
$W_{3,16}$	(4.76464,1.71080)	Sample	Success
$W_{3,17}$	(4.75160,0.978684)	Sample	Success
$W_{3,18}$	(4.07316,0.97440)	Not Sample	Type-II error
		Total path length	17.76424 m
		Total time taken	321 s
		Accuracy of exploration	73.68%

**Table 6:** The list of locations visited by the robot in trial-4 and sampling decision taken.

Waypoint	Location on Map	Decision	Result
$W_{4,0}$	(0.20626,3.84880)	Sample	Success
$W_{4,1}$	(0.20332,3.11855)	Sample	Success
$W_{4,2}$	(0.20837,2.39423)	Not Sample	Success
$W_{4,3}$	(0.20265,1.65911)	Sample	Success
$W_{4,4}$	(0.20471,0.93416)	Sample	Success
$W_{4,5}$	(0.20817,0.209653)	Sample	Success
$W_{4,6}$	(0.93839,0.208683)	Sample	type-I error
$W_{4,7}$	(1.66028,0.202513)	Sample	Success
$W_{4,8}$	(2.38826,0.204983)	Sample	Success
$W_{4,9}$	(3.12414,0.209353)	Not Sample	Success
$W_{4,10}$	(3.84809,0.208553)	Not Sample	type-II error
$W_{4,11}$	(4.57688,0.205633)	Sample	Success
$W_{4,12}$	(5.30855,0.205713)	Sample	Success
$W_{4,13}$	(5.30929,0.932024)	Not Sample	type-II error
$W_{4,14}$	(5.30382,1.659145)	Sample	Success
		Total path length	10.19308 m
		Total time taken	137 s
		Accuracy of exploration	80.0%

## Data Availability

Not applicable.

## References

[1] Truong N., Nisar T., Knox D., Prabhakar G. (2017). The influences of cleanliness and employee attributes on perceived service quality in restaurants in a developing country. Int. J. Cult. Tour. Hosp. Res..

[2] Cleaning a Nation: Cultivating a Healthy Living Environment. https://www.clc.gov.sg/research-publications/publications/urban-systems-studies/view/cleaning-a-nation-cultivating-a-healthy-living-environment.

[3] Cleaning Industry Analysis 2020-Cost & Trends. https://www.franchisehelp.com/industry-reports/cleaning-industry-analysis-2020-cost-trends/.

[4] Top Three Commercial Cleaning Trends in 2019. https://www.wilburncompany.com/top-three-commercial-cleaning-trends-in-2019/.

[5] Diab-El Schahawi M., Zingg W., Vos M., Humphreys H., Lopez-Cerero L., Fueszl A., Zahar J.R., Presterl E. (2021). Ultraviolet disinfection robots to improve hospital cleaning: Real promise or just a gimmick?. Antimicrob. Resist. Infect. Control.

[6] Chen J., Loeb S., Kim J.H. (2017). LED revolution: Fundamentals and prospects for UV disinfection applications. Environ. Sci. Water Res. Technol..

[7] Arnott B., Arnott M. (2018). Automatic Floor Cleaning Machine and Process.

[8] Martinovs A., Mezule L., Revalds R., Pizica V., Denisova V., Skudra A., Kolcs G., Zaicevs E., Juhna T. (2021). New device for air disinfection with a shielded UV radiation and ozone. Agron. Res..

[9] Dammkoehler D., Jin Z. (2017). Floor Cleaning Machine.

[10] Fleming M., Patrick A., Gryskevicz M., Masroor N., Hassmer L., Shimp K., Cooper K., Doll M., Stevens M., Bearman G. (2018). Deployment of a touchless ultraviolet light robot for terminal room disinfection: The importance of audit and feedback. Am. J. Infect. Control.

[11] Prabakaran V., Mohan R.E., Sivanantham V., Pathmakumar T., Kumar S.S. (2018). Tackling area coverage problems in a reconfigurable floor cleaning robot based on polyomino tiling theory. Appl. Sci..

[12] Muthugala M., Vega-Heredia M., Mohan R.E., Vishaal S.R. (2020). Design and control of a wall cleaning robot with adhesion-awareness. Symmetry.

[13] Sivanantham V., Le A.V., Shi Y., Elara M.R., Sheu B.J. (2021). Adaptive Floor Cleaning Strategy by Human Density Surveillance Mapping with a Reconfigurable Multi-Purpose Service Robot. Sensors.

[14] Chang C.L., Chang C.Y., Tang Z.Y., Chen S.T. (2018). High-efficiency automatic recharging mechanism for cleaning robot using multi-sensor. Sensors.

[15] Pathmakumar T., Sivanantham V., Anantha Padmanabha S.G., Elara M.R., Tun T.T. (2021). Towards an Optimal Footprint Based Area Coverage Strategy for a False-Ceiling Inspection Robot. Sensors.

[16] Giske L.A.L., Bjørlykhaug E., Løvdal T., Mork O.J. (2019). Experimental study of effectiveness of robotic cleaning for fish-processing plants. Food Control.

[17] Lewis T., Griffith C., Gallo M., Weinbren M. (2008). A modified ATP benchmark for evaluating the cleaning of some hospital environmental surfaces. J. Hosp. Infect..

[18] Asgharian R., Hamedani F.M., Heydari A. (2014). Step by step how to do cleaning validation. Int. J. Pharm. Life Sci..

[19] Malav S., Saxena N. (2018). Assessment of disinfection and cleaning validation in central laboratory, MBS hospital, Kota. J. Evol. Med Dent. Sci..

[20] Al-Hamad A., Maxwell S. (2008). How clean is clean? Proposed methods for hospital cleaning assessment. J. Hosp. Infect..

[21] Cloutman-Green E., D’Arcy N., Spratt D.A., Hartley J.C., Klein N. (2014). How clean is clean—Is a new microbiology standard required?. Am. J. Infect. Control.

[22] Pathmakumar T., Kalimuthu M., Elara M.R., Ramalingam B. (2021). An Autonomous Robot-Aided Auditing Scheme for Floor Cleaning. Sensors.

[23] Smart W.D., Kaelbling L.P. Effective reinforcement learning for mobile robots. Proceedings of the 2002 IEEE International Conference on Robotics and Automation (Cat. No. 02CH37292).

[24] Tai L., Paolo G., Liu M. Virtual-to-real deep reinforcement learning: Continuous control of mobile robots for mapless navigation. Proceedings of the 2017 IEEE/RSJ International Conference on Intelligent Robots and Systems (IROS).

[25] Rivera P., Valarezo Añazco E., Kim T.S. (2021). Object Manipulation with an Anthropomorphic Robotic Hand via Deep Reinforcement Learning with a Synergy Space of Natural Hand Poses. Sensors.

[26] Kozjek D., Malus A., Vrabič R. (2021). Reinforcement-Learning-Based Route Generation for Heavy-Traffic Autonomous Mobile Robot Systems. Sensors.

[27] Pi C.H., Dai Y.W., Hu K.C., Cheng S. (2021). General Purpose Low-Level Reinforcement Learning Control for Multi-Axis Rotor Aerial Vehicles. Sensors.

[28] Bing Z., Lemke C., Morin F.O., Jiang Z., Cheng L., Huang K., Knoll A. (2020). Perception-action coupling target tracking control for a snake robot via reinforcement learning. Front. Neurorobot..

[29] Schulman J., Wolski F., Dhariwal P., Radford A., Klimov O. (2017). Proximal policy optimization algorithms. arXiv.

[30] Wang D., Fan T., Han T., Pan J. (2020). A two-stage reinforcement learning approach for multi-UAV collision avoidance under imperfect sensing. IEEE Robot. Autom. Lett..

[31] Silver D., Lever G., Heess N., Degris T., Wierstra D., Riedmiller M. Deterministic policy gradient algorithms. Proceedings of the International Conference on Machine Learning, PMLR.

[32] Mousavi H.K., Liu G., Yuan W., Takáč M., Muñoz-Avila H., Motee N. (2019). A layered architecture for active perception: Image classification using deep reinforcement learning. arXiv.

[33] Hase H., Azampour M.F., Tirindelli M., Paschali M., Simson W., Fatemizadeh E., Navab N. Ultrasound-guided robotic navigation with deep reinforcement learning. Proceedings of the 2020 IEEE/RSJ International Conference on Intelligent Robots and Systems (IROS).

[34] Choi J., Park K., Kim M., Seok S. Deep reinforcement learning of navigation in a complex and crowded environment with a limited field of view. Proceedings of the 2019 International Conference on Robotics and Automation (ICRA).

[35] Pfeiffer M., Shukla S., Turchetta M., Cadena C., Krause A., Siegwart R., Nieto J. (2018). Reinforced imitation: Sample efficient deep reinforcement learning for mapless navigation by leveraging prior demonstrations. IEEE Robot. Autom. Lett..

[36] Niroui F., Zhang K., Kashino Z., Nejat G. (2019). Deep reinforcement learning robot for search and rescue applications: Exploration in unknown cluttered environments. IEEE Robot. Autom. Lett..

[37] Zuluaga J.G.C., Leidig J.P., Trefftz C., Wolffe G. Deep reinforcement learning for autonomous search and rescue. Proceedings of the NAECON 2018-IEEE National Aerospace and Electronics Conference.

[38] Hu J., Niu H., Carrasco J., Lennox B., Arvin F. (2020). Voronoi-based multi-robot autonomous exploration in unknown environments via deep reinforcement learning. IEEE Trans. Veh. Technol..

[39] Sampedro C., Rodriguez-Ramos A., Bavle H., Carrio A., de la Puente P., Campoy P. (2019). A fully-autonomous aerial robot for search and rescue applications in indoor environments using learning-based techniques. J. Intell. Robot. Syst..

[40] Wang Z., Bovik A.C., Sheikh H.R., Simoncelli E.P. (2004). Image quality assessment: From error visibility to structural similarity. IEEE Trans. Image Process..

[41] Alqaraawi A., Schuessler M., Weiß P., Costanza E., Berthouze N. Evaluating saliency map explanations for convolutional neural networks: A user study. Proceedings of the 25th International Conference on Intelligent User Interfaces.

[42] Yoo S., Jeong S., Kim S., Jang Y. (2021). Saliency-Based Gaze Visualization for Eye Movement Analysis. Sensors.

[43] Schulman J., Moritz P., Levine S., Jordan M., Abbeel P. (2015). High-dimensional continuous control using generalized advantage estimation. arXiv.

[44] Karlik B., Olgac A.V. (2011). Performance analysis of various activation functions in generalized MLP architectures of neural networks. Int. J. Artif. Intell. Expert Syst..

[45] Liang E., Liaw R., Nishihara R., Moritz P., Fox R., Gonzalez J., Goldberg K., Stoica I. (2017). Ray rllib: A composable and scalable reinforcement learning library. arXiv.

[46] Liang E., Liaw R., Nishihara R., Moritz P., Fox R., Goldberg K., Gonzalez J., Jordan M., Stoica I. RLlib: Abstractions for distributed reinforcement learning. Proceedings of the International Conference on Machine Learning, PMLR.

[47] Ketkar N. (2017). Introduction to pytorch. Deep Learning with Python.

[48] Brockman G., Cheung V., Pettersson L., Schneider J., Schulman J., Tang J., Zaremba W. (2016). Openai gym. arXiv.

[49] Moritz P., Nishihara R., Wang S., Tumanov A., Liaw R., Liang E., Elibol M., Yang Z., Paul W., Jordan M.I. Ray: A distributed framework for emerging AI applications. Proceedings of the 13th USENIX Symposium on Operating Systems Design and Implementation (OSDI 18).

[50] Quigley M., Conley K., Gerkey B., Faust J., Foote T., Leibs J., Wheeler R., Ng A.Y. (2009). ROS: An open-source Robot Operating System. IEEE International Conference on Robotics and Automation (ICRA) Workshop on Open Source Software.

[51] Marin-Plaza P., Hussein A., Martin D., Escalera A.D.L. (2018). Global and local path planning study in a ROS-based research platform for autonomous vehicles. J. Adv. Transp..

[52] Fox D., Burgard W., Thrun S. (1997). The dynamic window approach to collision avoidance. IEEE Robot. Autom. Mag..

